# Laboratory assessment of molluscicidal and cercariacidal effects of *Glinus lotoides* fruits

**DOI:** 10.1186/1756-0500-7-220

**Published:** 2014-04-08

**Authors:** Gebrehiwot Kiros, Berhanu Erko, Mirutse Giday, Yalemtsehay Mekonnen

**Affiliations:** 1 Wollo University, P.O. Box 1145, Dessie, Ethiopia; 2 Aklilu Lemma Institute of Pathobiology, Addis Ababa University, P.O. Box 1176, Addis Ababa, Ethiopia; 3 College of Natural and Computational Sciences, Microbial, Cellular and Molecular Biology Department, Addis Ababa University, P.O. Box 1176, Addis Ababa, Ethiopia

**Keywords:** *Glinus lotoides*, Molluscicide, Cercariacide, Schistosomiasis, Ethiopia

## Abstract

**Background:**

The negative impact of synthetic molluscicides on the environment and their high cost necessitated search for an alternative approach of using plant extracts for the control of schistosomiasis. The objective of this study was, therefore, to evaluate aqueous and ethyl acetate crude extracts of *Glinus lotoides* fruits for their cercariacidal activity and molluscicidal effect against schistosome snail intermediate hosts.

**Methods:**

Assessment of the molluscicidal activity against *Biomphalaria pfeifferi* was made by immersion method in accordance with WHO guideline. The results of mortality were statistically analyzed using probit analysis. The attenuating effect of the plant on *Schistosoma mansoni* cercariae was determined using establishment of adult worms as a parasitological parameter post exposure.

**Results:**

The 24 and 48 hour-LC_50_ values for the aqueous extract of *G. lotoides* fruits were 47.1 and 44.1 mg/L, respectively, whereas that of ethyl acetate were 66.1 and 59.6 mg/L, respectively. The 24 and 48 hour LC_90_ values for the aqueous extract of *G. lotoides* fruits were 56.96 and 51.0 mg/L, respectively, while that of ethyl acetate were 77.2 and 70.0 mg/L, respectively. The *in vitro* cercariacidal activity was determined after 2 hrs of exposure to the aqueous plant extract. It was found out that the LC_50_ and LC_90_ values were 18.7 and 41.7 mg/L, respectively. Besides, infectivity of *Schistosoma mansoni* cercariae to mice was determined by exposing mice to cercariae pre-treated with the sub-lethal concentrations (3.7, 11.6 and 18.7 mg/L) of the aqueous extract. A significant reduction in worm burden in mice was obtained at 11.6 mg/L (p < 0.05). Moreover, the reduction in number of worms recovered was highly significant at 18.7 mg/L (p < 0.001).

**Conclusions:**

The results showed that *G. lotoides* has molluscicidal activity against *B. pfeifferi* snails and cercariacidal activity against *S. mansoni*. Yet, further comprehensive evaluation is recommended for the possible use of *G. lotoides* against *B. pfeifferi* and the schistosome parasite.

## Background

Schistosomiasis is a parasitic disease caused by blood-flukes of the genus *Schistosoma*. People get infected by having contact with freshwater that harbors free-swimming larval forms of the parasite shed from freshwater snail intermediate hosts [1,2]. Globally, about 200 million people are infected with schistosomes and about 600 million are at risk [3]. Schistosome infection is the second parasitic infection after malaria as a cause of morbidity and mortality [4,5].

In Ethiopia, two forms of human schistosomiasis have been known to be endemic. Intestinal schistosomiasis is caused by *Schistosoma mansoni* and is transmitted by *Biomphalaria* snails whereas urinary schistosomiasis is caused by *Schistosoma haematobium* and is transmitted by bulinid snails [6]. It is estimated that four million people are affected by schistosomiasis and 29.89 million people are placed at risk of infection [7].

Although effective, safe, and inexpensive single-dose praziquantel is available to treat schistosomiasis with the prime objective of morbidity control [4], sustainable control of schistosomiasis requires integrated approach including repeated mass chemotherapy using praziquantel, public health education focusing on behaviour changes towards risk factors, improving sanitation, provision of clean water supply, and snail control [8]. Since there is a possibility of re-infection even after repeated mass chemotherapy with praziquantel [9], complementing chemotherapy with measures such as snail control is necessary to prevent re-infection of people in endemic areas.

Snail control through the use of synthetic molluscicides forms an important part in the integrated schistosomiasis control programme [10]. However, the high cost of synthetic compounds such as Bayluscide® (Bayer, Leverkusen, Bayerwerk, Germany) and their negative impact on the environment have stimulated interest in search for alternative molluscicides of plant origin [11]. Many plant species have been proved to have molluscicidal properties against different snail species. The most potent plant that has been known to have molluscicidal activity against snail intermediate hosts of schistosome is *Phytolacca dodecandra*[12]. *Alternanthera sesselis*[13], *Balanites aegyptiaca*[14] and *Jatropha curcas*[15] also lie among plants with molluscicidal activities.

*Glinus lotoides*, a plant indigenous to Ethiopia, has a variety of traditional medicinal uses including the treatment of tapeworm infestation and other intestinal parasites, using as anti-fungal agent and for relieving of inflammation, and also as a remedy for diarrhea. Four triterpinoid saponins and flavonoides have been isolated from the seeds of *G. lotoides* grown in Ethiopia [16]. Pharmacological studies on *G. lotoides* seeds showed *in vitro* taenicidal activity against *Taenia saginata*[17] and the *in vitro* and *in vivo* taenicidal activity against *Hymenolopis nana*[18,19]. The methanol extract of *G. lotoides* seeds of the plant also possess antimicrobial activity [20]. Besides, the hydro-alcoholic seed extract of the plant was also reported to have anti-cancer activity. However, no work has been reported so far on the molluscicidal and cercariacidal activity of *G. lotoides* in Ethiopia. This study, therefore, aimed to evaluating the aqueous and ethyl acetate extracts of *G. lotoides* fruits for their molluscicidal and cercariacidal activities.

## Methods

### Plant material collection

Fresh fruits of *G. lotoides* were collected from its natural habitat around Koka Water Reservoir, located at 90 km southeast of Addis Ababa, in Oromia Regional State, Ethiopia. Fruits were then transported to laboratory in a sack to avoid direct sunlight. Identity of the plant was confirmed by the third author at Aklilu Lemma Institute of Pathobiology, Addis Ababa University (AAU) and a voucher specimen (Kiros 001) was deposited at the National Herbarium, College of Natural Sciences, Addis Ababa University, Ethiopia.

### Preparation and extraction of the plant material

The fruits of *G. lotoides* were washed using tap water, and air dried at room temperature (25-27°C) for two weeks. After drying, the fruits were ground into powder with the help of pestle and mortar. Powder was sieved using a mesh of 250 microns to get fine material. The powder was then kept in a screw caped glass container until extraction [21]. Extraction of the fine powdered material was made using two solvents; water and ethyl acetate. Aqueous extract was prepared by soaking 1 g of the dried plant powder in 300 ml of aged water and shaking it for 24 hours at 160 rpm at room temperature (25°C). After shaking, the unfiltered suspension was directly used to prepare a stock solution. Ethyl acetate extract on the other hand was prepared by soaking 10 g of the dried fruits powder in 250 ml of ethyl acetate. The solution was extracted by shaking it for 72 hours at 160 rpm at room temperature. The extracted material was then concentrated to dryness under reduced pressure using Rota vapor at 45°C. The crude extract obtained was then refrigerated at -20 ºC until bioassay [14,22].

### Preparation of the stock solution and serial dilution

For aqueous extract, the plant material soaked for 24 hours was directly used to prepare the stock solution by adjusting it to 1000 ml using aged water. For the ethyl acetate extract, stock solution was prepared by dissolving 1 g up to 1000 mg/L concentration by adding a suitable volume of aged water. For each extract, further serial dilutions to which snails were exposed were made from the stock solution. Once the extent of the toxicity range was determined, several convenient concentrations of the stock solution were prepared to give mortalities between 0 to 100%. Serial dilutions for water extract were prepared to a final concentration of 0.0, 40, 42.5, 45, 47.5, 50, 52.5, 55, 57.5 and 60 mg/L. Similarly, the final concentrations for ethyl acetate extract were 0.0, 57.5, 60, 62.5, 65, 67.5, 70, 72.5, 75, 77.5 and 80 mg/L. In both cases, the series of concentrations were prepared in a final volume of 250 ml aged water. These concentrations were prepared by taking the calculated volume from the stock solution and then diluted with aged water until1000 ml [22,23].

### Molluscicidal potency test of the plant extracts

The snail species used in this study was *Biomphalaria pfeifferi*. The snails were collected from their natural habitats, in small streams of Sheno and Chacha, which are located at 80 and 100 km North of Addis Ababa, respectively. Uniform sized (8-12 mm) snails were collected using dip-net scoop and were brought to Aklilu Lemma Institute of Pathobiology (ALIPB) laboratory using open plastic buckets which contained some sand, vegetation and water. Snails were cleaned from debris attached to them and screened for any trematode infection according to Christensen and Frandsen [24]. The molluscicidal potency test of *G. lotoides* crude extract against *B. pfeifferi* was performed according to established procedure [25]. A total of 10 snails were exposed per test to different concentrations of aqueous (37.5-60 mg/L) and ethyl acetate (55–80 mg/L) extracts. Snails were exposed to the extract for 24 hours under room temperature (25-27°C). After 24 hours, snails were removed from the container and were transferred to a new recipient filled with aged water. The snails then remained under observation for another 24 hours. A group of ten snails were kept in aged water under the same experimental conditions as a negative control group. The experiment for each concentration was repeated three times. The snails were not fed during the course of the experiment. It had been observed that healthy snails live up to 5 days or more without food, provided other environmental conditions are constant [10]. Mortality rate was recorded after every 24 hours up to 48 hours. Toxicity was expressed as LC_50_ and LC_90_ corresponding to concentrations that killed 50% and 90% of the tested snails, respectively. The LC_50_ and LC_90_ values were determined for both 24 and 48 hours exposure [26,27]. Snails were considered dead if they did not move and were either retracted well into or hanging out of the shell, with discolored body. Mortality was also assessed by probing the snails with a blunt wooden probe to elicit typical withdrawal movements.

### Mice infectivity of *Schistosoma mansoni* cercariae pretreated with sublethal concentrations of *Glinus lotoides* fruit aqueous extract

Establishment of adult worms 48 days post-exposure was used as a parasitological parameter to measure the attenuating effect of the plant on *S. mansoni* cercariae. Infectivity of *S. mansoni* cercariae to mice was determined by pre-exposing the cercariae to sublethal concentrations. Cercarial sublethal concentrations (LC_15_, LC_35_ and LC_50_) were determined using probit analysis computer software programme. These were found to be 3.7, 11.6 and 18.7 mg/L. Aqueous extract of *G. lotoides* fruit was adjusted to stock solution of 1000 mg/ml by aged water. A series of concentrations containing 3.7, 11.6 and 18.7 mg/L were prepared from the stock solution. Five test tubes were prepared for every concentration. One hundred fifty cercariae (the pre-determined infective dose) were transferred using micro-pipette into each test tube. The cercariae were then exposed to these concentrations for 2 h at room temperature before being used to infect mice. Five mice of both sexes with the age of 5–7 weeks were individually exposed for every concentration for 1 hour using the tail immersion method in a total volume of 10 ml of cercarial suspension [28]. Mice were washed to remove cercariae that may remain in hairs. Then, the numbers of cercariae left in the test tubes were counted, and the cercariae to which the mice exposed were determined.

Each mouse was exposed to 128 ± 10 cercariae. After 2 hour exposure to the fruit extract, mice were maintained under standard condition in the animal house. As a control, five mice were individually exposed to the same number of cercariae untreated to the fruit extract. At 45 days post infection, fecal samples for each mouse was collected and checked for schistosome eggs using KatoKatz smear. At 48 days post infection, all mice were dissected. Worms were recovered from liver and intestine by perfusion using saline solution. Number of worms recovered was counted for both experimental and control groups. Then worm load per mouse was determined as described by Al-Zanbagi and Abuljadayel [29] and Kamel et al. [30]. Percentage reduction in worm burden resulting from the plant extract was also calculated using the following formula as described by Metwally [31]. P = (C-V)/C X100; where: P = % reduction in worm burden, V = mean number of worms recovered from mice infected with pretreated cercariae (experimental), C = mean number of worms recovered from mice infected with untreated cercariae (infected control).

### Cercariacidal potency test of the aqueous extract of *Glinus lotoides* fruits

#### *Preparation of cercarial suspension*

Infected *B. pfeifferi* snails were collected from Bishan Gari, which is located at 235 km southeast of Addis Ababa. Snails were individually placed in a Petri dish containing 5 ml of water followed by exposure to artificial light for 2 hours. Emerging cercariae were observed under dissecting microscope. The cercariae shed by the snails were then pooled into a glass beaker and mixed well to have uniform distribution. The number of cercariae in 200 μl was counted under microscope using replicated samples. The volume of water to get 150 cercariae was then estimated. This was used for the *in vivo* test. For the *in vitro* test, the cercariae in 100 μl were counted using replicated samples and the average was used.

#### *In vitro cercariacidal activity test of the aqueous extract of Glinus lotoides fruits*

A series of concentrations containing 5, 10, 20, 40 and 55 mg/L were prepared in a Petri dish. An average of twenty freshly shed cercariae were transferred into each Petri dish using micro-pipette. The same number of cercariae was placed in Petri dishes containing aged water as a control group. Each dilution, as well as control group, was tested in duplicate. The cercariae were observed under a dissecting microscope for survival and mortality at a successive interval of 30 minutes, 1 hour, 1 and half hours and 2 hours. They were considered to be dead when they stopped movement, sank down and detached their tail [27]. At the end of each experiment, iodine was added for clarity in counting of the total number of cercariae as a confirmation of accuracy of the counting procedure. The LC_50_ and LC_90_ values of aqueous extract of *G. lotoides* fruits on *Schistosoma mansoni* cercariae after 2 hour exposure were determined [15].

### Statistical analysis

The LC_50_ and LC_90_ were determined by probit analysis using SPSS version 20 with 95% confidence limit. Number of adult worms recovered from mice was represented as mean ± SD of the mean. Data was subjected to ANOVA to determine the significant difference in worm burden between the experimental and control mice [32]. A p-value of < 0.05 was set as statistically significant.

## Results

Aqueous and ethyl acetate extracts of *G. lotoides* fruits showed activities against *B. pfeifferi* and *S. mansoni* cercariae. The extracts caused concentration dependent mortality of *B. pfeifferi* from 0 - 100% mortality in concentrations of 37.5-80 mg/L. The 24 and 48-hour- LC_50_ and LC_90_ values of aqueous extract of *G. lotoides* fruit were higher than that of ethyl acetate (Table 1).

**Table 1:** **The molluscicidal effect of aqueous and Ethyl acetate extracts of *Glinus lotoides *** **fruits against *Biomphalaria pfeifferi***

**Exposure period**	**Plant product**	**Lethal concentration (mg/l) or mg/L**		**Negative control (% mortality)**
		**LC**_**50**_	**LC**_**90**_	
**24 hour**	aqueous extract	47.1 CI (44.6-49.6)	56.96 CI (53.9-62.6)	0
	Ethyl acetate extract	66.1 CI (66.52-68.51)	77.2 CI (73.8-83.2)	
**48 hour**	aqueous extract	44.1 CI (41.9-55.9)	51 CI (48.8-54.9)	0
	Ethyl acetate extract	59.6 CI (55.8-62.1)	70 (67.1-77.2)	

*In vitro* cercariacidal activity test for the aqueous extract of *G. lotoides* fruits showed that the plant possessed cercariacidal activity against *S. mansoni* cercariae. The mortality was both time and concentration dependent. At the first 30 minutes of exposure to 5 mg/L, no mortality of *S. mansoni* cercariae was observed. However, after 2 hour exposure, mortality at the same concentration was elevated to 20%. The LC_50_ and LC_90_ values were determined after 2 hours exposure to the plant extract (Table 2).

**Table 2:** ***In vitro *** **cercariacidal effect of aqueous extract of *Glinus lotoides *** **fruits**

**Exposure period**	**Plant product**	**Lethal concentrations (mg/l) or mg/L**		**Negative control (% mortality)**
		**LC**_**50**_	**LC**_**90**_	
**2 hour**	Aqueous extract	18.7 CI (14.8-22.5)	41.7 CI (35.998-50.50)	0

Infectivity of *S. mansoni* cercariae to mice was significantly reduced (P < 0.05) by exposing mice to cercariae pre treated with the sub lethal concentrations of the aqueous extract. Pre-exposure of the cercariae to 3.7, 11.6 and 18.7 mg/L reduced the establishment of adult worms in the liver and intestine by 35.3%, 55.9% and 91.2%, respectively (Table 3). Comparison of the mean worm burden per mouse for each group using one way ANOVA showed that there was no statistically significant difference between mice infected with untreated cercariae and mice infected with cercariae pre-exposed to the lower concentration, 3.7 mg/L (P > 0.05). In contrast, significant difference was obtained in mice exposed to cercariae pre-treated with 11.6 mg/L (P < 0.05). Moreover, pre-exposure of the cercariae to the highest concentration almost inhibits the establishment of adult worms, which was highly significant (P < 0.001). At this concentration, the infectivity of the cercariae and their subsequent development into adult worms was highly suppressed with worm recovery reduced from 6.8 worms/mouse in the control group to 0.6 worm/mouse at the highest concentration.

**Table 3:** **Percentage reduction of worm burden in mice infected with cercariae pre-treated with aqueous extract of *G. lotoides *** **fruits**

**Worm burden (Mean ± SD)**					
**Treatments**	**Male**	**Female**	**Couple**	**Total**	**Total worm burden reduction (%)**
	**M ± SD**	**M ± SD**	**M ± SD**	**M ± SD**	
Infected control	2.4 ± 0.418	3.2 ± 0.636	1.2 ± 0.398	6.8 ± 0.398	-
3. 7 mg/L	1.4 ± 0.328	2.2 ± 0.48	0.8 ± 0.35	4.4 ± 0.5	35.3%
11.6 mg/L	1.8 ± 0.79*	0.8 ± 0.21*	0.4 ± 0.17*	3 ± 0.247*	55.9%
18.7 mg/L	0.4 ± 0.08**	0.2 ± 0.09**	0.0 ± 0.0**	0.6 ± 0.178**	91.2%

Note Results are expressed as mean ± SD of the mean of five observations. *Indicates the level of significance of the experimental mice compared to the infected control. No statistical significant difference was obtained at 3.7 mg/L (P > 0.05), *statistically significant at 11.6 mg/L (P < 0.05) and **highly significant difference at 18.7 mg/L (P < 0.001) compared with infected control.

## Discussion

The results of the present study revealed that *Biomphalaria pfeifferi* was susceptible to the fruits of *G. lotoides* extracts at different concentrations. The LC_50_ and LC_90_ values for the aqueous extract after 24 hours exposure were 47.1 and 56.96 mg/L, respectively. The LC_50_ and LC_90_ of the same extract after 48 hours exposure were 44.1 and 51 mg/L, respectively. The LC_50_ and LC_90_ for the ethyl acetate extract after 24 hour exposure were 66.1 and 77.2 mg/L, respectively. However, after 48 hours exposure, the extract produced LC_50_ and LC_90_ of 59.6 and 70 mg/L respectively. The potency of *G. lotoides* aqueous extract on *B. pfeifferi* in the present study was much higher when compared to other related plant extracts used in other work. Diallo et al. [33] reported that the aqueous extract of *Glinus oppositifolius* showed 100% mortality of *B. Pfeifferi* only at 200 mg/L.

The continuous crawling out from the test extract solution of snails exposed to sub lethal concentration is taken as an irritative and avoidance behavior as described by Evans et al. [34] and Brackenbury [35] for bulinid snails. The ability of the snails to crawl out of the test extract solution and then aggregate at the water-air interface in snails exposed to sub-lethal concentration, where their body is not in close and continuous contact to the plant extract, is similar to observation described by Adetunji and Salawu [10]. Azare et al. [13] also observed movement of snails to the side of the container in an attempt to escape from the test media containing *Althernanthera sessils* treated water. The mechanism of *B. pfeifferi* partially leaving the treated water has been found to increase the survival of *B. straminae* exposed to sub lethal doses of niclosamide [36] and *B. glabrata* exposed to *Phytolacca dodecandra*[37].

Phytochemical studies of plants reported for their molluscicidal activities in different countries revealed the presence of saponins, alkaloids and flavonoides [38,39]. Saponins are also one of the major classes of compounds that possess molluscicidal activity. *Phytolacca dodecandra,* one of the potent molluscicidal plants, has saponins. Endale et al. [16] demonstrated that the seeds of *G. lotoides* contain saponins and flavonoides.

As it was evident from the LC_50_ and LC_90_ values of the 24 and 48 hours exposure, the molluscicidal potency of the aqueous extract was higher than that of the ethyl acetate. This variation in activity might be attributed to the fact that the saponins and flavonoides responsible for its activity are extracted in greater measures with more polar solvent such as water than with less polar solvents such as ethyl acetate. This finding corroborated other studies that reported differences in the activities of extracts obtained from the same plant part using different solvents. For instance, the aqueous extract of *Nerium indicum* is more potent than the N-butanol extract of the same plant [40].

For a plant to be considered as a candidate molluscicide, a crude preparation of its parts should be active at 100 mg/L or less and kill 90% of snails exposed for 24 h [25,41]. In the present study, aqueous extract of *G. lotoides* fruit was active at a concentration as low as 56.96 mg/L and killed 90% of the snails within 24 hour contact to the plant extract. Thus, *G. lotoides* fruit aqueous extract can be considered as a candidate molluscicide. On the other hand, the alcoholic or lipophilic plant extract is considered as a candidate molluscicide if it has LC_90_ values equal or less than 20 mg/l after 24 hours. In the present study, ethyl acetate extract of *G. lotoides* fruit showed LC_90_ value of 70 mg/L after 24 exposures to the ethyl acetate extract. Therefore, according to this criterion, ethyl acetate extract may not be considered as a candidate molluscicide.

The present observation showed that aqueous extract of *G.lotoides* fruits possess cercariacidal activity against *S. mansoni* cercariae. The activity was both time and dose dependent. This result is similar to that of Mohamed et al. [42] who reported the cercariacidal activity of the crushed seed of *Nigella sativa* to be both time and concentration dependent. This finding is also in line with the reported data for *Milletia thoningii* by Perret et al. [43] and *Irish germanica* by Singaba et al. [23] who reported the time-concentration relationship of the plants extracts*.* Other studies by Rug and Rupel [15] using different solvent extracts of *Jatropha curcas* and by Ahmed and Ramzy [44] using aqueous extract of *Solanum nigrum* also reported similar findings.

Aqueous extract of *G. lotoides* fruits were significantly reduced the infectivity of *S. mansoni* cercariae on mice. At lower concentration (3.7 mg/L) the worm load per mouse was decreased by 35.3% compared with control group, while at the highest concentration (18.7 mg/L), this was reduced by 91.2%. According to the present results, the plant was effective in this respect at the highest concentration, 18.7 mg/L. The decrease in worm load/mouse could be due to the attenuation of cercarial mobility and activity to be unable to penetrate the mice skin during exposure to such cercariae [30].

*G. lotoides* is an indigenous plant grown in Ethiopia. It is traditionally known for the treatment of tape worm infestation and other intestinal worms [45-47], diarrhea, as a purgative as well as for curing boils and wounds [48]. In the current study, *G. lotoides* was proved to have molluscicidal and cercariacidal activity. Since its anthelminthic activities have been established [16], this new finding would widen the spectrum of its medicinal values.

## Conclusions

From the present study, it can be concluded that aqueous and ethyl acetate crude extracts of *G. lotoides* fruits possess molluscicidal properties against the intermediate snail host, *B. pfeifferi.* Both extracts showed differing molluscicidal activity. *B. pfeifferi* was found to be more susceptible to the aqueous extract of *G. lotoides* fruits than to that of ethyl acetate. The aqueous extract also showed *in vitro* cercariacidal property against *S. mansoni* cercariae. Moreover, pre-treatment of *S. mansoni* cercariae with the aqueous extract of *G. lotoides* fruits before mice infection has produced a significant reduction of the infectivity of cercariae to mice. However, bioactivity guided fractionation to isolate the specific compound responsible for the molluscicidal as well as cercariacidal activity of the extracts is recommended.

## Competing interests

The authors declare that they have no competing interests.

## Authors’ contributions

GK: wrote the first draft of the proposal, collected samples, conducted the experiment, analyzed the data and wrote the first draft of the manuscript. BE: come up with research idea, read and polished the proposal, participated in the collection of snail samples, supervised the conduct of the laboratory experiments, and participated in the write-up of the manuscript. MG: contributed to the development of the research idea, read and improved the proposal, collected and identified plant samples, supervised laboratory experiments and read and participated in the write up of the manuscript. YM: participated in the development of the proposal and writ-up of the manuscript. All authors read and approved the final manuscript.
